# Egr3 Dependent Sympathetic Target Tissue Innervation in the Absence of Neuron Death

**DOI:** 10.1371/journal.pone.0025696

**Published:** 2011-09-28

**Authors:** Lin Li, Laurie C. Eldredge, David H. Quach, Avinash Honasoge, Katherine Gruner, Warren G. Tourtellotte

**Affiliations:** 1 Department of Pathology, Feinberg School of Medicine, Northwestern University, Chicago, Illinois, United States of America; 2 Division of Neuropathology, Department of Pathology, Feinberg School of Medicine, Northwestern University, Chicago, Illinois, United States of America; 3 Department of Neurology, Feinberg School of Medicine, Northwestern University, Chicago, Illinois, United States of America; University of Cincinnatti, United States of America

## Abstract

Nerve Growth Factor (NGF) is a target tissue derived neurotrophin required for normal sympathetic neuron survival and target tissue innervation. NGF signaling regulates gene expression in sympathetic neurons, which in turn mediates critical aspects of neuron survival, axon extension and terminal axon branching during sympathetic nervous system (SNS) development. Egr3 is a transcription factor regulated by NGF signaling in sympathetic neurons that is essential for normal SNS development. Germline Egr3-deficient mice have physiologic dysautonomia characterized by apoptotic sympathetic neuron death and abnormal innervation to many target tissues. The extent to which sympathetic innervation abnormalities in the absence of Egr3 is caused by altered innervation or by neuron death during development is unknown. Using Bax-deficient mice to abrogate apoptotic sympathetic neuron death *in vivo*, we show that Egr3 has an essential role in target tissue innervation in the absence of neuron death. Sympathetic target tissue innervation is abnormal in many target tissues in the absence of neuron death, and like NGF, Egr3 also appears to effect target tissue innervation heterogeneously. In some tissues, such as heart, spleen, bowel, kidney, pineal gland and the eye, Egr3 is essential for normal innervation, whereas in other tissues such as lung, stomach, pancreas and liver, Egr3 appears to have little role in innervation. Moreover, in salivary glands and heart, two tissues where Egr3 has an essential role in sympathetic innervation, NGF and NT-3 are expressed normally in the absence of Egr3 indicating that abnormal target tissue innervation is not due to deregulation of these neurotrophins in target tissues. Taken together, these results clearly demonstrate a role for Egr3 in mediating sympathetic target tissue innervation that is independent of neuron survival or neurotrophin deregulation.

## Introduction

The sympathetic nervous system (SNS) is a division of the autonomic nervous system that has essential roles in maintaining tissue and organ homeostasis. A detailed understanding of the mechanisms involved in establishing and maintaining normal sympathetic target tissue innervation is of considerable interest because abnormal development and degeneration of the SNS is a component of many human neurological diseases. A variety of molecules involved in different stages of SNS development have been identified. For example, migration of neural crest cells from the margins of the dorsal neural tube, which give rise to postganglionic sympathetic neurons, is dependent on many diffusible factors and their cognate receptor signaling including neuregulin-1/ErbB [1], GFRα3/Ret [2], [3] and semaphorin 3a/neuropilin 1 [4]. In addition, specification of the post-migratory sympathetic precursors is induced by diffusible factors such as the bone morphogenetic proteins (BMPs) and a variety of transcriptional regulators including Mash1, Phox2a and b, Hand2 (dHand), and Gata3 that regulate acquisition of a noradrenergic phenotype [5], . To establish connections in the periphery, postganglionic sympathetic neurons depend on vascular derived diffusible factors such as Artemin [8] and neurotrophin 3 (NT-3) [9] to guide axons along blood vessels to reach their targets. One of the most important target tissue-derived trophic factors is nerve growth factor (NGF), which is essential for sympathetic neuron survival and target tissue innervation [10], [11].

NGF has been known for many decades to be essential for sympathetic neuron survival [12], [13], but only more recently has a particularly important role in target tissue innervation also been appreciated in vivo [10]. For example, loss of NGF with concurrent loss of the pro-apoptosis molecule Bax, leads to abnormal sympathetic target tissue innervation in the context of inhibited neuron death [10], whereas overexpression of NGF in target tissues leads to hyperinnervation [14], [15]. Early NGF signaling events within sympathetic neurons depend upon binding of NGF to the TrkA tyrosine kinase receptor and signaling through the Ras/MAPK pathway to mediate some aspects of NGF-dependent neuronal differentiation [16], [17]. However, very little is known about how this signaling pathway orchestrates new gene expression within sympathetic neurons and what role it may have in regulating axon pathfinding and target tissue innervation in vivo.

Egr3 is a transcriptional regulator that is induced in sympathetic neurons by NGF, its expression is coupled to Ras/MAPK signaling, and germline Egr3-deficient mice have physiologic dysautonomia characterized by apoptotic sympathetic neuron death and abnormal target tissue innervation [18]. Since sympathetic neuron death occurs concurrently with abnormal target tissue innervation in Egr3-deficient mice, it has not been clear to what extent Egr3 has a direct role in target tissue innervation relative to its role in neuron survival in vivo. In this study, we examined whether Egr3 has a role in target tissue innervation using an in vivo context that prevented sympathetic neuron death. In the absence of the pro-apoptosis protein Bax, sympathetic neuron death was prevented [19], [20] and mice with simultaneous loss of Bax and Egr3 had atrophic sympathetic neurons, persistent dysautonomia and sympathetic target tissue innervation abnormalities. Target tissue innervation was not effected uniformly, with some tissues showing severe sympathetic innervation abnormalities, while in other tissues no innervation abnormalities were observed. These findings are similar to results obtained in NGF/Bax double knockout mice [10] and are consistent with previous studies indicating that Egr3 acts as an effector of NGF signaling in sympathetic neurons to mediate normal target tissue innervation [18].

## Results

### Egr3-deficient sympathetic neurons are protected from death in the absence of Bax

**Figure 1:**
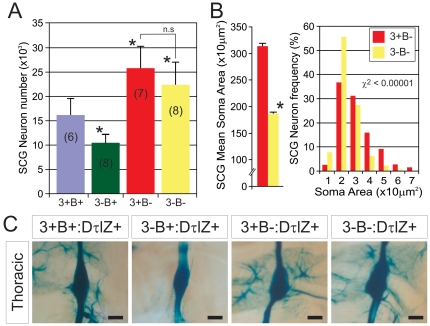
Target tissue innervation abnormalities in the absence of sympathetic neuron death in Egr3-deficient mice. (**A**) In the absence of the pro-apoptotic molecule Bax, sympathetic neurons in the SCG were protected from both physiologic and Egr3-dependent death. Approximately 1/3 of SCG neurons were lost in Egr3-deficient (3-B+) compared to wild type (3+B+) mice. In the absence of Bax, physiologic sympathetic neuron death (3+B−) and Egr3-dependent neuron death (3-B−) was inhibited. Inhibition of physiologic death increased the total number of SCG neurons as previously reported [10]. (number of SCG analyzed from 8–12 week old mice indicated in parentheses; *  =  p<0.01 and n.s. =  non-significant compared to 3+B+ mice, Student's t test) (**B**) In the absence of Egr3 and inhibited apoptosis (3-B−), SCG neurons were significantly atrophic. (8–12 week old mice analyzed, *  =  p<0.001 compared to 3+B−, Student's t test and size-frequency, χ^2^<0.0001). (**C**) Similar qualitative results were observed in thoracic prevertebral ganglia as apoptotic death was rescued in all sympathetic neurons. Compared to wild type ganglia (3+B+), 3-B+ ganglia were notably smaller and ganglia lacking Bax, with or without loss of Egr3 were larger. All mice were 8-12 weeks of age and expressed the DτlZ sympathetic reporter transgene to highlight the ganglia after LacZ histochemical staining (scale bar  =  25 µm).

Previous studies demonstrated that approximately 1/3 of sympathetic neurons die by apoptosis shortly after birth in the absence of Egr3 and that neuron loss was correlated with decreased sympathetic target tissue innervation and physiologic dysautonomia [18]. However, it is not known whether dysautonomia in the absence of Egr3 is due to failure of sympathetic neurons to properly innervate target tissues, neuron death or a combination of the two processes. To address whether Egr3 has a role in target tissue innervation independent of neuron death that occurs in Egr3-deficient mice, we mated them to Bax knockout mice [20]. In the absence of the pro-apoptotic protein Bax, sympathetic neurons are refractory to growth factor-dependent physiologic death and they do not require neurotrophins, such as NGF or NT-3 for survival during development [19], [21]. Thus, it was possible to block both Egr3-dependent and physiologic sympathetic neuron death to ask whether target tissue innervation is still disrupted in the absence of Egr3. To confirm that physiologic and Egr3-dependent (pathologic) cell death could be rescued in mice lacking both Egr3 and Bax (3-B−), we quantified the number of sympathetic neurons within the SCG from adult wild type (3+B+), Egr3^−/−^; Bax^+/+^ (3-B+), Egr3^+/+^; Bax^−/−^ (3+B−), and Egr3^−/−^; Bax^−/−^ (3-B−) mice using optical dissector neuron counting and unbiased stereology. In this genetic background, 3-B+ mice had approximately 1/3 fewer SCG neurons compared to wild type (3+B+) mice, similar to previously published results [18]. Bax-deficient (3+B-) mice had a supra-physiologic number of SCG neurons compared to wild type (3+B+) as a consequence of inhibited physiologic death, also in agreement with previously published results [10] (Fig. 1A). However, in the absence of both Bax and Egr3 (3-B−), physiologic and Egr3-dependent neuron loss was inhibited similar to Bax-deficient (3+B−) mice. Similar changes were observed in thoracic sympathetic ganglia from mice that also expressed the DτlZ sympathetic reporter transgene. For example, compared to wild type (3+B+) ganglia, 3-B+ ganglia were smaller, whereas ganglia from mice lacking Bax (3+B− and 3-B−) were notably larger (Fig. 1C). Thus, loss of Bax protects neurons from death in the absence of Egr3, similar to the results observed for sympathetic and sensory neurons that are protected from death by loss of Bax in the absence of the neurotrophins NGF and NT-3 [10], [21].

Target tissue-derived NGF has an essential role in sympathetic neuron survival and it has a trophic role in their differentiation and neurite outgrowth during development. In the absence of Bax, sympathetic neurons do not require NGF for survival, but they continue to respond to NGF by undergoing cellular hypertrophy and extending neurites [19], [22]. Consistent with our previous results indicating that Egr3 is a transcriptional effector of NGF signaling [18], sympathetic neurons devoid of both Bax and Egr3 (3-B−) in vivo were significantly atrophic compared to Bax-deficient (3+B−) neurons (Fig. 1B) (χ^2^<0.00001). These results indicate that Egr3 mediates at least some trophic responses of sympathetic neurons that are similar to NGF function [10], [19].

### Sympathetic target tissue innervation abnormalities persist in Egr3-deficient mice despite inhibited sympathetic neuron death

**Figure 2:**
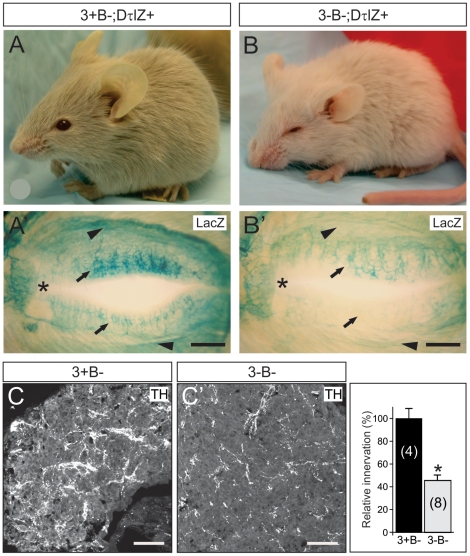
Disruption of sympathetic target tissue innervation originating from the SCG in the absence of neuron death in Egr3-deficient mice. (**A**) Compared to 8 week old Bax-deficient control mice (3+B−) (**B**) physiologic ptosis and (**B**') innervation abnormalities to the eye were present in the absence of Egr3 and Bax (3-B−). Results show lacZ staining in mice also expressing the DτlZ sympathetic reporter transgene which highlighted (**B**') superior tarsus muscle innervation (arrowheads) and meibomian gland (arrows) innervation abnormalities to the eyes in 3-B− mice compared to (**A**') 3+B− control mice. (* = nasal canthus; scale bar = 100 µm). (**C, C**') Sympathetic innervation to the pineal gland, a major target of SCG neurons, was reduced by >50% in 3-B− mice compared to 3+B− control mice (4–8 weeks old). (scale bar = 200 µm)

In the absence of Bax, it was possible to examine whether Egr3 has a role in sympathetic target tissue innervation independent of concurrent neuron death. Egr3-deficient mice have blepharoptosis which is a physiological sign of abnormal sympathetic innervation from the SCG neurons to the eye and a failure to properly elevate upper and lower eyelids [18]. We found that blepharoptosis was present in 3-B− mice despite rescue of sympathetic neuron death in the SCG (Fig. 2A, B). Using DτlZ transgenic reporter mice to label sympathetic neurons and their innervating axons it was possible to examine target tissue innervation with high resolution. Sympathetic innervation to the superior and inferior tarsus muscles (Fig. 2A' and B', arrowheads) and to meibomian glands on the inner surface of the eyelid (Fig. 2A' and B', arrows) were clearly abnormal in 3-B− mice (Fig. 2B') compared to 3+B− mice (Fig. 2A'), similar to previous results obtained from Egr3-deficient mice where there is concurrent sympathetic neuron death [18].

**Figure 3:**
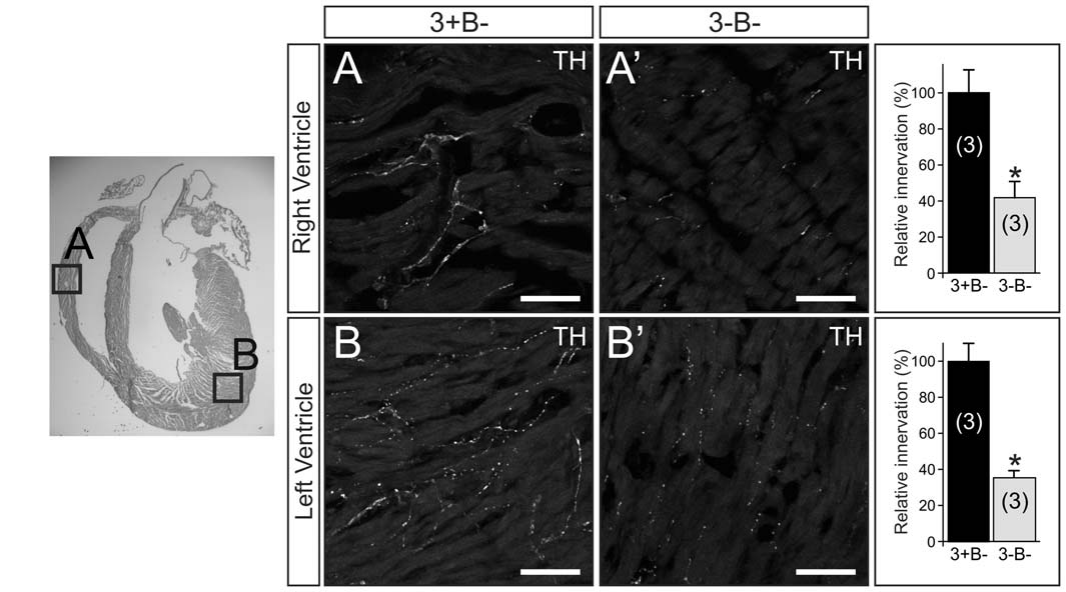
Cardiac sympathetic innervation abnormalities in the absence of Egr3 and sympathetic neuron death. Sympathetic innervation to the (**A**) right and (**B**) left ventricles of the heart was reduced by 58% and 65%, respectively in (**A**'**, B**'') 3-B− mice compared to (**A, B**) 3+B− mice. (scale bar = 50 µm) (results of TH immunofluorescence quantification representing the mean ± standard error; * = p<0.01, Student's t test compared to 3+B− mice; number of 4–8 week old animals analyzed indicated in parentheses).

**Figure 4:**
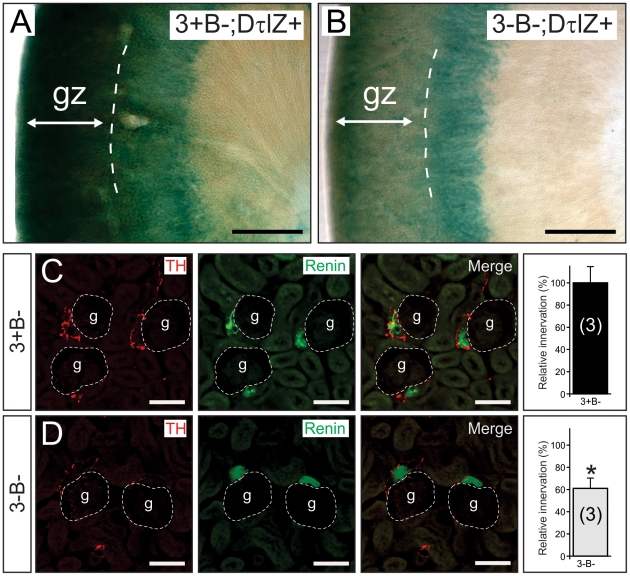
Renal sympathetic innervation abnormalities in the absence of Egr3 and sympathetic neuron death. (**A, B**) Lacz histochemistry highlighted diffuse renal cortical sympathetic innervation in mice expressing the DτlZ reporter transgene. (**B**) In the absence of Egr3 and sympathetic neuron death (3-B−;DτlZ) there was a marked decrease in lacZ staining in the glomerular zone (gz) of the renal cortex compared to (**A**) control (3+B−; DτlZ) mice. (scale bar = 500 µm) (**C, D**) The decreased sympathetic innervation in the gz was correlated with a 40% reduction of (**C, D**) sympathetic innervation (TH immunofluorescence, left) to the renin expressing juxtaglomerular (jg) apparatus (renin immunofluorescence, middle) within the gz. (scale bar = 50 µm) (results of TH immunofluorescence quantification representing the mean ± standard error in the area juxtaposed to the jg apparatus; * = p<0.01, Student's t test compared to 3+B−; DτlZ+ control mice; number of 4–8 week old animals analyzed indicated in parentheses).

**Figure 5:**
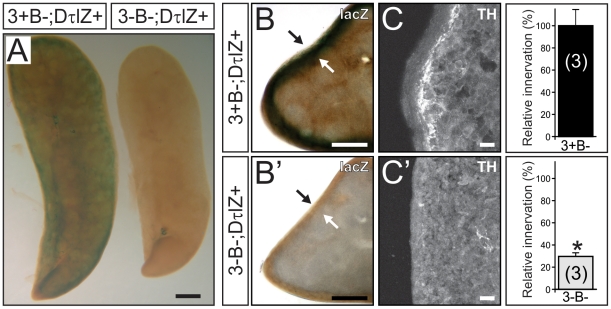
Abnormal innervation to the spleen in the absence of Egr3 and sympathetic neuron death. (**A**) Sympathetic innervation to the spleen was highly diminished in Egr3-deficient mice (3-B−; DτlZ+) compared to control (3+B−; DτlZ+) mice. Note the markedly decreased lacZ reaction product in the Egr3-deficient spleen (right compared to left) which correlated with decreased subcapsular (**B**') lacZ reaction (arrows) and (**C**') TH+ terminal axon innervation in Egr3-deficient mice (3-B−; DτlZ+) relative to (**B, C**) control (3+B−; DτlZ+) mice. In Egr3-deficient spleens sympathetic innervation was reduced by >70%. (results of TH immunofluorescence quantification representing the mean ± standard error from 3 4–8 week old mice of each genotype; * = p<0.0001, Student's t test; scale bar = A, 1 mm; B, B', 0.5 mm; C, C', 10 µm).

Using tyrosine hydroxylase (TH) immunofluorescence and semi-quantitative densitometry, we examined target tissue innervation arising from neurons in several sympathetic ganglia. For example, similar to the eye, sympathetic innervation from the SCG to the pineal gland was reduced by 55% in the absence of Egr3 and neuron death (3-B−; Fig. 3C') compared to Bax-deficient (3+B−; Fig. 3C) mice. In the heart, where sympathetic innervation primarily arises from sympathetic neurons within the para-vertebral stellate ganglion (STG), innervation to the right (Fig. 3A, A') and left (Fig. 3B, B') ventricles was reduced by 58% and 65%, respectively. Similarly, sympathetic innervation from pre-vertebral celiac ganglion neurons was markedly decreased in the glomerular zone (gz) of the renal cortex from 3-B− mice compared to 3+B− mice as demonstrated by the DτlZ transgene and lacz histochemistry (Fig. 4A, B). Diminished renal cortical innervation was correlated with a 40% reduction in sympathetic innervation to the renin-secreting juxtaglomerular apparatus in 3-B− relative to 3+B- kidneys (Fig. 4C, D). Sympathetic innervation to the spleen, which also arises primarily from the pre-vertebral celiac ganglion, was significantly decreased in 3-B− relative to 3+B− mice, whether examined qualitatively with the DτlZ reporter transgene and lacZ histochemistry (Fig. 5A, B, B') or using semi-quantitative TH immunofluorescence to demonstrate a 70% decrease in subcapsular innervation (Fig. 5C, C').

**Figure 6:**
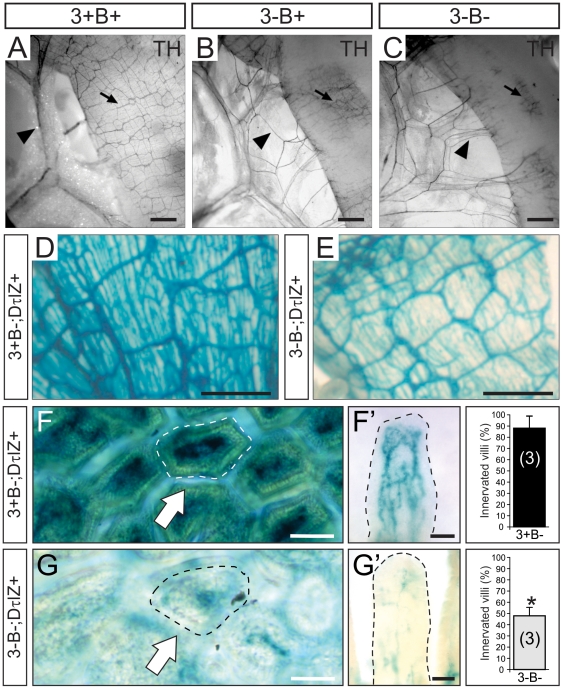
Abnormal sympathetic innervation to small bowel in the absence of Egr3 and sympathetic neuron death. (**A**) in wild type (3+B+) mice, tyrosine hydroxylase (TH) immunohistochemistry performed on vascularized intact small bowel demonstrated sympathetic axons coursing along the mesenteric blood vessels (arrowhead) and prominent intercrossing pattern of TH+ fibers in the bowel wall (arrow). (**B, C**) In preparations from mice lacking Egr3, with (3-B+) our without Bax (3-B−), the TH+ axons were noted to prematurely defasciculate from the mesenteric blood vessels prior to innervating the bowel wall (arrowhead) and the pattern of intercrossing TH+ fibers in the bowel wall was highly attenuated (arrow) (4 week old mice shown). (**D, E**) The attenuated mural innervation was confirmed using DτlZ sympathetic reporter mice which showed a highly attenuated innervation of the bowel wall in 3-B− relative to 3+B− mice. (**F**) Sympathetic innervation to the small bowel mucosa was observed in the vascular core of mucosal villi from control (3+B−; DτlZ+) sympathetic reporter mice (arrow, villi shown in whole mount preparation in the axial plane). (**F**') Sympathetic innervation within the small bowel villi paralleled the vascular capillary network (villus shown in whole mount preparation in the longitudinal plane). (**G**) In Egr3-deficient mice with inhibited sympathetic neuron death (3-B−; DτlZ+) <45% of the villi were innervated (arrow) and (**G**') most villi that did have some innervation showed a highly attenuated abnormal innervation pattern. (quantitative results represent the mean ± standard error of >500 villi scored; * = p<0.01, Student's t test compared to 3+B−; DτlZ+ control mice; scale bar = 50 µm; number of 4–8 week old animals analyzed indicated in parentheses).

Sympathetic innervation to the small bowel which arises primarily from pre-vertebral mesenteric sympathetic ganglia was also highly disrupted in 3-B− and 3-B+ relative to 3+B+ mice. Using TH immunohistochemistry on intact vascularized small bowel, sympathetic innervation was visualized coursing along the mesenteric vessels (Fig. 6A, arrowhead) to innervate the bowel wall (Fig. 6A, arrow). By contrast, innervation to the small bowel in both 3-B+ and 3-B− mice appeared to defasciculate and branch away from the larger blood vessels prior to innervating the bowel (Fig. 6B, C, arrowheads) and the complex intercrossing pattern of TH+ fibers within the bowel wall was markedly abnormal (Fig. 6B, C, arrows). The TH staining pattern in the bowel wall was variable in different preparations as a result of inqdequate antibody penetration in some adult tissues. However, the results were confirmed using LacZ histochemistry and DτlZ+ mice which showed a markedly attenuated mural pattern of DβH+ fibers in 3-B− (Fig. 6E) relative to 3+B- mice (Fig 6D). When the DτlZ sympathetic reporter transgene was used to examine sympathetic innervation in to the small bowel in greater detail, more than 50% of the mucosal villi had no appreciable innervation in 3-B− mice (Fig. 6F, G, arrows). In most of the remaining villi, innervation was detectable but showed highly attenuated branching and decreased innervation complexity within the villus core (Fig. 6F', G').

**Figure 7:**
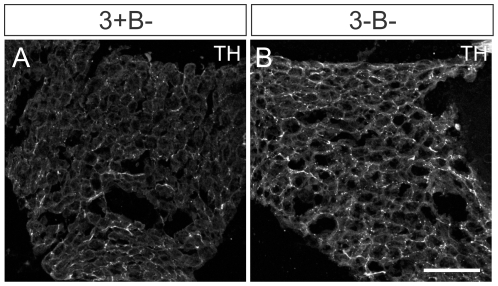
Normal sympathetic innervation was observed in some tissues in the absence of Egr3 and neuron death. In brown fat for example, which receives dense sympathetic innervation to regulate thermogenesis no definitive changes were observed in (**A**) 3+B− relative to (**B**) 3-B− mice. (scale bar  = 100 µm).

While many tissues showed significant innervation abnormalities using semi-quantitative TH immunofluorescence and/or terminal axon staining with the DτlZ reporter transgene in 3-B− mice, some tissues such as thymus, lung, stomach, pancreas, liver and brown fat (Fig. 7) showed no definitive changes in innervation. In addition, some tissues such as trachea and esophagus showed only mild innervation abnormalities (Table 1). Similar heterogeneous effects on sympathetic target tissue innervation were also previously observed in mice lacking both NGF and Bax [10].

**Table 1:** Target tissue innervation in 3-B− mice compared to 3+B− mice.

Heart	+ (p<0.001)
Spleen	+ (p<0.001)
Distal Ileum	+ (p<0.001)
Eyelid (tarsus muscles and meibomian glands)	+
Pineal Gland	++ (p<0.001)
Kidney	++ (p<0.001)
Trachea	+++
Esophagus	+++
Sympathetic chain ganglia	+++
Intercostal nerves (proximal projections)	+++
Thymus	++++
Lung	++++
Stomach	++++
Pancreas (head)	++++
Liver	++++
Brown fat	++++

+  =  highly reduced innervation relative to adult 3+B− control mice.

+++ and ++  =  intermediate innervation relative to adult 3+B− control mice.

++++  =  similar innervation relative to adult 3+B− control mice.

p values, Student's t test, from semi-quantitative TH immunofluorescence.

### Target tissue innervation abnormalities are not due to deregulation of NGF or NT-3 expression in target tissues in the absence of Egr3

**Figure 8:**
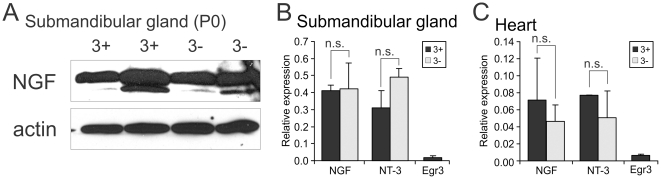
Normal NGF and NT-3 expression in two target tissues with markedly abnormal sympathetic innervation in Egr3-deficient mice. (**A**) Western blotting for NGF and actin (loading control) showed that NGF protein levels were similar in submandibular glands between wild type (3+) and Egr3-deficient (3-) mice (submandibular gland protein lysates from two different newborn mice shown). qPCR to measure the level of NGF and NT-3 transcript expression in (**B**) submandibular gland and (**C**) heart showed no significant differences between 3+ and 3- mice. Egr3 expression in both tissues was low and near detection threshold. (results represent the mean ± standard error of tissues from 3–4 animals 8–10 weeks old of each genotype; n.s.  =  non-significant, Student's t test).

NGF is expressed by target tissues where it is essential for sympathetic neuron survival and target tissue innervation [11]. NT-3 is expressed in blood vessels along which sympathetic axons travel and in some target tissues where it has a particularly important role in early SNS development, primarily through TrkA receptor signaling [23]. Thus, it is possible that loss of Egr3 in the germline could deregulate the expression of these neurotrophins in the periphery and at least partially explain the sympathetic innervation abnormalities observed in mice lacking Egr3. However, in the submandibular gland, an organ that contains very high levels of NGF and robust sympathetic innervation highly effected by loss of Egr3 [18], there was no evidence that NGF-β protein levels were altered in 3- compared to 3+ submandibular glands (Fig. 8A). Similarly, no changes in the levels of either NGF-β or NT-3 mRNA expression were identified in submandibular gland (Fig. 8B) or heart which also receives robust sympathetic innervation that is altered in Egr3-deficient mice (Fig. 8C). In both tissues, Egr3 was expressed at a very low level near the detection threshold for the qPCR assay. Thus, abnormal innervation in Egr3-deficient mice occurs in the context of normal expression of NGF and NT-3 in target tissues, indicating that Egr3 has no role in regulating their expression in the periphery.

## Discussion

NGF is produced and released by target tissues where it mediates sympathetic neuron survival and innervation during development, a concept that has been clearly established in mice lacking NGF or its cognate tyrosine kinase receptor TrkA (NTRK1) [10], [11], [24]. Egr3 is regulated by NGF/TrkA signaling in sympathetic neurons and while previous studies indicate that it does not have a direct role in their survival like NGF, it appears to be required for normal terminal axon extension, branching and target tissue innervation. Although some sympathetic neurons die in the absence of Egr3, our current results indicate that neuron death is a consequence, rather than a cause of abnormal target tissue innervation. We previously showed that Egr3 does not have a direct role in sympathetic neuron survival in vitro [18] and here we demonstrated that when sympathetic neuron death is prevented in vivo by the absence of the pro-apoptotic protein Bax, target tissue innervation abnormalities persist in the absence of Egr3. Although the sympathetic neurons that would otherwise die in the absence of Egr3 are most likely not normal in the Bax null genetic background, sensory neurons that lack both TrkA signaling and Bax are still able to generate limited innervation in the central nervous system but not to peripheral target tissues [22]. Similar to previous studies with mice lacking both NGF and Bax [10], we found no evidence that rescue of sympathetic neuron death in the Bax null background also rescued target tissue innervation, indicating that any Bax null neurons that would have otherwise died in the absence of Egr3 were also not competent to innervate their targets. Thus, persistent abnormal innervation despite neuron survival indicates that Egr3 has role separate from neuron survival and that neuron death in vivo is likely a consequence of inadequate acquisition of target-derived NGF for a population of sympathetic neurons that have relatively impaired target innervation. However, since the absence of Egr3 leads to only partial loss of sympathetic innervation, Egr3 appears to have a role in regulating the extent of sympathetic target tissue innervation rather than permitting it to occur. This is consistent with the observation that Egr3 has no major role in NGF-dependent neurite outgrowth or branching in vitro (data not shown) and with our previous in vivo results which indicated that Egr3 has a role in late stages of SNS development, when terminal axons are extending and branching within target tissues, rather than during early axon outgrowth [18].

In similar studies performed using Bax/NGF double knockout mice or sympathetic neurons isolated from Bax-deficient mice devoid of NGF treatment in vitro, terminal axon extension and innervation abnormalities were observed to differing degrees in target tissues [10] and atrophy due to decreased neuron metabolism was observed [19], respectively. We observed strikingly similar abnormalities in Bax/Egr3 double knockout mice which had significant sympathetic neuron atrophy and heterogeneous effects on sympathetic innervation to various target tissues. While it is possible that Egr3 regulates NGF (or NT3) expression in peripheral tissues, we did not find any evidence that they were abnormally expressed in the absence of Egr3. Rather, we have previously suggestedthat Egr3 is an effector of NGF signaling within sympathetic neurons that regulates essential genes required for normal tissue innervation. This hypothesis was further supported by this study which showed similar abnormalities present in the SNS between Bax/NGF and Bax/Egr3 double mutant mice. However, there were some differences between Bax/NGF and Bax/Egr3 double knockout mice. For example, in some tissues such as submandibular gland, parotid gland and heart, NGF appears to be necessary for target tissue innervation. By contrast, the absence of Egr3 leads to attenuation of the innervation to these organs rather than complete loss. Similarly, in some tissues such as thymus, lung, stomach and liver, NGF appears to have a major role in their innervation, whereas Egr3 does not. These discrepancies could reflect that Egr3 is just one of a number of effectors of NGF signaling and therefore it could have a relatively restricted function in a sub-population of NGF-dependent neurons. In addition, Bax/NGF knockout mice die at birth, despite rescued sympathetic neuron death, making it impossible to rule out developmental delay as a cause of the discrepancies or to study the end-stage target tissue innervation phenotypes in adult animals as we did for Bax/Egr3 knockout mice. Nevertheless, considering previous studies showing that Egr3 is regulated by NGF signaling in sympathetic neurons, it is reasonable to conclude that Egr3 modulates gene expression required for at least some aspects of NGF-mediated sympathetic terminal axon extension and target tissue innervation [18].

Currently, the specific target genes regulated by Egr3 during sympathetic target tissue innervation are not known. One clue related to the mechanisms of Egr3 action in sympathetic neurons may come from the observation that axons prematurely defasciculate from the major mesenteric vessels as they innervate the bowel in the absence of Egr3. Perhaps genes that regulate axon fasciculation and/or axon guidance along blood vessels may be involved. This seems plausible as axon guidance molecules expressed by neurons such as neuropilin-1 (npn1) have a major role in axon fasciculation and target tissue innervation patterning [25]. Future studies will be required to identify the precise target genes regulated by Egr3 to better understand how it participates in orchestrating NGF signaling during target tissue innervation.

NGF has an essential role in regulating sympathetic neuron target tissue innervation that is in part regulated by Egr3. Although the current results indicate that Egr3 does not regulate NGF or NT-3 in peripheral tissues, it has not been formally demonstrated that Egr3 has its primary function within sympathetic neurons to impact their target tissue innervation. A formal proof that Egr3 has an essential role in regulating target gene expression within sympathetic neurons to mediate target tissue innervation, and potentially to maintain innervation homeostasis, will require more sophisticated genetic studies to demonstrate its sympathetic neuron autonomous function.

## Materials and Methods

### Ethics statement

All experiments with animals reported complied with relevant national and international guidelines. All experimental procedures involving mice complied with a animal studies protocol (#2009-1886) approved by the Northwestern University Institutional Animal Care and Use Committee (IACUC) and PHS animal welfare assurance to Northwestern University (#A3283-01).

### Animals

Mice with a germline deletion of Egr3 (Egr3^−/−^) and Bax (Bax^−/−^) were previously described and backcrossed >5 generations into the C57BL/6J isogenic background [20], [26]. The DβH-τlacZ (DτlZ) sympathetic reporter transgenic mice, which express axon localized β-galactosidase (τlacZ) in noradrenergic neuron (including sympathetic neurons) were generated on a B6SJL genetic background and backcrossed >4 generations into the C57BL/6J isogenic background as previously described [18].

Genotyping was performed by PCR using genomic DNA isolated from tail biopsy tissue. The sequences of the genotyping primers and PCR conditions are available upon request. Adolescent and adult mice (4–20 weeks old) were used for all analyses with the exception that newborn mice were used to obtain protein lysates from submandibular gland for Western blotting. For most experiments littermate or age-matched wild type mice were used as control. In experiments using Egr3/Bax double knockout (3-B−) mice, littermate mice lacking only Bax expression (3+B−) were used as control to examine the effects of loss of Egr3 on SNS development in the context of inhibited neuron death [19]. Three or more animals per genotype were analyzed per experimental condition as indicated.

### Tissue preparation

Anesthetized mice were perfused through the heart with 0.1 M phosphate buffered (pH = 7.2) 4% paraformaldehyde (PFA) and tissues were post-fixed at 4°C for 1–4 hours. Some tissues were cryoprotected overnight at 4°C in graded (15–30%) sucrose, embedded in OCT and 12 µm frozen sections were analyzed. For some experiments, tissues were isolated fresh for protein and/or RNA isolation. For quantitative studies involving neuron counts and morphometry, PFA fixed tissues were embedded in paraffin and serial sectioned at 16 µm.

### Ganglion neuron counts and evaluation of neuron soma area

SCG neuron numbers from 8–12 week old Egr3^+/+^; Bax^+/+^ (3+B+), Egr3^−/−^; Bax^+/+^ (3-B+), Egr3^+/+^; Bax^−/−^ (3+B−), and Egr3^−/−^; Bax^−/−^ (3-B−) mice were determined using unbiased stereology and optical dissector methods (StereoInvestigator, Microbrightfield, Williston, VT) on every fifth serial section as previously described [18], [27]. Neurons within randomly selected stereologic counting frames in the SCG with clear nuclear and nucleolar profiles were analyzed. SCG neuron soma area was estimated using Metamorph software (Molecular Devices, Sunnyvale, CA) on calibrated digital photomicrographs. Neurons from multiple sections throughout the rostral-caudal extent of the SCG were counted and the soma area of >300 neurons per genotype was measured.

### Immunohistochemistry

Immunofluorescence staining for tyrosine hydroxylase (TH; sheep anti-TH, 1∶5000 Chemicon) to identify sympathetic terminal axon innervation and Renin (goat anti-renin, 1∶1000 Santa Cruz) to identify the juxtaglomerular apparatus in kidney was performed on frozen tissue sections. A secondary antibody conjugated with Cy3 (Jackson Immunoresearch) or Alexa-488 (Invitrogen) was used to localize primary antibody binding. In some experiments tissues were stained intact without sectioning using TH primary antibody and immunoperoxidase detection as previously described [3]. In all experiments, non-immune serum was used in place of the primary antibody to control for non-specific antibody binding.

### Western Blotting

Western blotting was performed as previously described [28]. Primary antibodies to detect β-NGF (Sigma, N6655, 1∶1000) and actin (Santa Cruz, sc-1616, 1∶1000) were used with HRP-conjugated secondary antibodies (Jackson Immunoresearch) and enhanced chemiluminescence (ECL) detection (Amersham) on total cellular protein isolated from Egr3^+/+^ (3+) and Egr3^−/−^ (3-) newborn (P0) submandibular glands.

### Semi-quantitative sympathetic axon terminal innervation

Quantification of target tissue innervation was performed using fluorescence densitometry: Fluorescent images were captured with a Zeiss LS510 confocal microscope using identical aperture and photomultiplier tube voltage settings to ensure accurate comparison between tissues from 3+B- and 3-B- mice. The density of TH-positive terminals was calculated using MetaMorph software as the ratio of total immunofluorescence in a specified area and averaged for 6 separate confocal frames at 200x total magnification. Representative sympathetic target tissues, such as heart (left and right ventricles), distal ileum, trachea, esophagus, stomach, thymus, pineal gland, lung, pancreas, liver, spleen and kidney were analyzed in areas with the highest density of axons identified in control (3+B−) tissues. The “relative innervation” in 3-B− mice was expressed as a mean percent of control (3+B−) from 3–8 animals of each genotype as indicated.

### LacZ enzyme histochemistry

Whole mount lacZ enzyme histochemistry of periorbital tissue, SCG, kidney, spleen and distal ileum from 4–8 week old 3+B− and 3-B− mice that also carried the DτlZ sympathetic reporter transgene was performed as previously described [18]. Innervation to intestinal villi from similar portions of the distal ileum was quantified in 3+B−;DτlZ+ and 3-B−;DτlZ+ mice. The adventitia and deep muscularis was removed from the lacZ stained ilea and the mucosal surface was photographed using a Nikon E600 microscope and digital camera (RT-Slider, Diagnostic Instruments, Sterling Heights, MI). For each genotype, >500 villi were scored to determine whether they contained innervation or not based upon visualization of lacZ reaction product within each villus core. Villi containing any identifiable innervation were scored as “innervated” and those without visible innervation were scored as “non-innervated.” The results were reported as the mean % of innervated villi compared to the total number of villi examined from 3 mice of each genotype.

### Semi-quantitative real-time PCR (qPCR)

qPCR was performed as previously described in detail [27]. Briefly, total cellular RNA was isolated from wild type 8–10 week old SCG and two sympathetic target tissues, submandibular gland and heart. cDNA was synthesized using Superscript III reverse transcriptase (Invitrogen) and random octomer/oligo-dT priming according to recommendations by the manufacturer. Relative gene expression was determined using Sybr Green (Invitrogen) fluorescence with non-intron spanning primers. Standard curves were generated for each primer pair using mouse genomic DNA and the target gene expression results were normalized to GAPDH expression for each sample analyzed. The results were expressed either as unitless expression relative to GAPDH or fold-change expression relative to control tissue. Oligonucleotide primers amplified the following nucleotide sequences: **GAPDH**: (Genbank NM_008084, nt 202–627), **NGF**: (Genbank NM_013609, nt 604–904), **NT-3**: (Genbank NM_008742, nt 446–846) and **Egr3**: (Genbank NM_018781, nt 163–563).
